# A Prospective Randomized Controlled Trial of the Effects of Vitamin D Supplementation on Cardiovascular Disease Risk

**DOI:** 10.1371/journal.pone.0036617

**Published:** 2012-05-07

**Authors:** Adam D. Gepner, Rekha Ramamurthy, Diane C. Krueger, Claudia E. Korcarz, Neil Binkley, James H. Stein

**Affiliations:** University of Wisconsin School of Medicine and Public Health, Madison, Wisconsin, United States of America; New York University School of Medicine, United States of America

## Abstract

Vitamin D (VitD) supplementation has been advocated for cardiovascular risk reduction; however, supporting data are sparse. The objective of this study was to determine whether VitD supplementation reduces cardiovascular risk. Subjects in this prospective, randomized, double-blind, placebo-controlled trial of post-menopausal women with serum 25-hydroxyvitamin D concentrations >10 and <60 ng/mL were randomized to Vitamin D3 2500 IU or placebo, daily for 4 months. Primary endpoints were changes in brachial artery flow-mediated vasodilation (FMD), carotid-femoral pulse wave velocity (PWV), and aortic augmentation index (AIx). The 114 subjects were mean (standard deviation) 63.9 (3.0) years old with a 25-hydroxyvitamin D level of 31.3 (10.6) ng/mL. Low VitD (<30 ng/mL) was present in 47% and was associated with higher body-mass index, systolic blood pressure, glucose, CRP, and lower FMD (all p<0.05). After 4 months, 25-hydroxyvitamin D levels increased by 15.7 (9.3) ng/mL on vitamin D3 vs. −0.2 (6.1) ng/mL on placebo (p<0.001). There were no significant differences between groups in changes in FMD (0.3 [3.4] vs. 0.3 [2.6] %, p = 0.77), PWV (0.00 [1.06] vs. 0.05 [0.92] m/s, p = 0.65), AIx (2.7 [6.3] vs. 0.9 [5.6] %, p = 0.10), or CRP (0.3 [1.9] vs. 0.3 [4.2] mg/L, p = 0.97). Multivariable models showed no significant interactions between treatment group and low VitD status (<30 ng/mL) for changes in FMD (p = 0.65), PWV (p = 0.93), AIx (p = 0.97), or CRP (p = 0.26).In conclusion, VitD supplementation did not improve endothelial function, arterial stiffness, or inflammation. These observations do not support use of VitD supplementation to reduce cardiovascular disease risk.

**Trial Registration:**

ClinicalTrials.gov **NCT00690417**

**Trial Registration:**

ClinicalTrials.gov **NCT01049048**

## Introduction

Although the definition of “low” vitamin D (VitD) status is controversial, suboptimal VitD status is common worldwide [1]. Circulating 25-hydroxyvitamin D [25(OH)D] values <30 ng/mL are present in up to 57% of healthy US adults and in up to 50% of postmenopausal women with osteoporosis [1], [2]. A growing literature suggests that low levels of VitD are associated with increased total mortality [3] and cardiovascular disease (CVD) risk [4]–[10]. VitD inadequacy has been linked to hypertension, insulin resistance, metabolic syndrome, and congestive heart failure [4]–[8]; however, these associations mostly are derived from cross-sectional and observational studies [4]–[8], [11]–[13]. Low VitD status could increase CVD risk by activating a pro-inflammatory cascade resulting in endothelial dysfunction and increased arterial stiffness, markers that contribute to hypertension and that are well-recognized surrogates of CVD risk [14]–[17]. The limited number of interventional studies that investigated the effects of VitD supplementation on CVD risk have had mixed results [15], [18]–[20]. To date, the only prospective randomized trial that evaluated the effects of VitD supplementation on CVD events was the Women's Health Initiative [18]. No differences in CVD events or stroke over 7 years were observed in women treated with 400 IU daily of VitD compared to placebo; however, this study has been criticized for using an inadequate VitD dose [18]. Based on currently available data, this dose of VitD only would be expected to raise 25(OH)D levels by 2–3 ng/mL [21]. Moreover, the compliance in WHI only was about 60%, so this small increase in 25(OH)D levels would have even less of an observed effect [18]. Observational data relating low VitD to CVD risk and other health conditions have led some medical providers to prescribe VitD supplements for CVD risk reduction, although data supporting this intervention are sparse. The aim of this study was to determine if VitD supplementation would improve flow-mediated vasodilation (FMD) of the brachial artery, a measure of endothelial function, and 2 measures of arterial stiffness, carotid-to-femoral pulse wave velocity (PWV) and aortic augmentation index (AIx). These markers, and their changes, are predictors of CVD risk [14], [16], [17].

## Methods

The protocol for this trial, CONSORT checklist, and Supplemental Table S1 are available as supporting information; see Checklist S1, Protocol S1, and Table S1.

**Figure 1:**
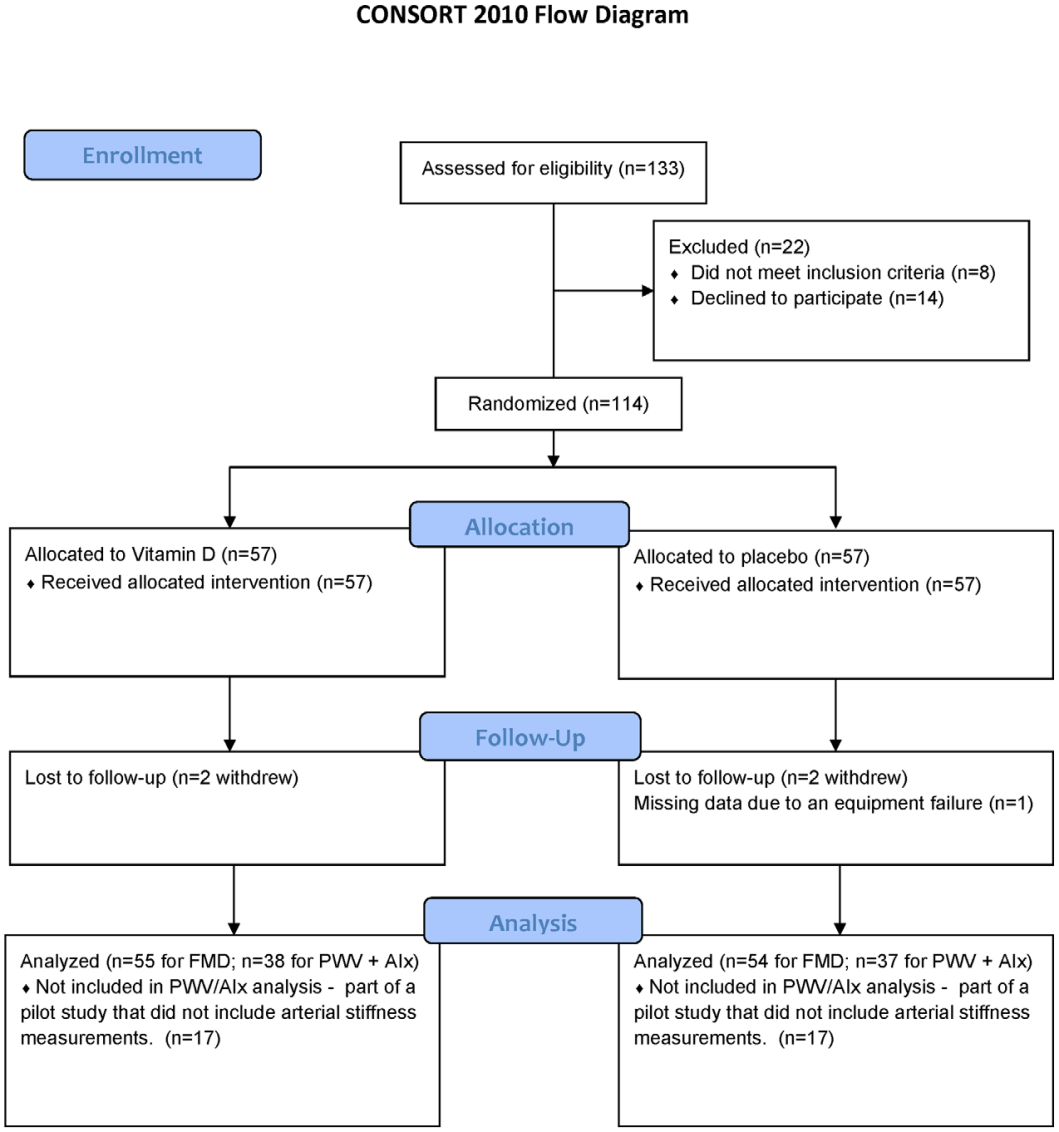
CONSORT 2010 Flow Diagram. The first 34 subjects were part of a pilot study performed under NCT 00690417. The remainder of the subjects were performed under NCT 01049048.

**Table 1:** Baseline Subject Characteristics.

	Placebo (N = 57)	Vitamin D (N = 57)	P-value between treatment groups
**Age (years)**	63.6 (3.1)	64.1 (3.0)	0.419
**Years since menopause**	13.4 (5.1)	14.1 (6.5)	0.921
**Height (cm)**	164.7 (5.1)	162.7 (6.8)	0.083
**Weight (lbs)**	151.0 (27.8)	157.7 (27.4)	0.114
**Body-mass index (kg/m^2^)**	25.3 (5.1)	27.1 (4.7)	0.022
**Total 25OH vitamin D (ng/mL)**	32.3 (10.5)	30.3 (10.7)	0.353
**Calcium (mg/dL)**	9.4 (0.3)	9.4 (0.4)	0.862
**Glucose (mg/dL)**	97.4 (27.0)	97.5 (33.5)	0.978
**Total cholesterol (mg/dL)**	202.9 (36.2)	205.3 (31.0)	0.650
**Triglycerides (mg/dL)**	95.8 (47.4)	102.0 (50.2)	0.496
**HDL cholesterol (mg/dL)**	70.9 (20.3)	68.5 (18.5)	0.516
**LDL cholesterol (mg/dL)**	112.9 (32.9)	116.5 (28.0)	0.462
**Total/HDL cholesterol ratio**	3.1 (0.9)	3.2 (0.8)	0.563
**C-reactive protein (mg/L)**	1.9 (2.9)	2.5 (2.9)	0.287
**Brachial artery diameter (cm)**	0.36 (0.04)	0.36 (0.05)	0.664
**Absolute FMD (cm)**	0.016 (0.018)	0.018 (0.011)	0.521
**Maximum Relative FMD (%)**	4.57 (3.30)	5.05 (3.38)	0.452
**Heart rate (bpm)**	59.7 (9.6)	56.9 (7.0)	0.157
**Brachial SBP (mmHg)**	122.2 (11.8)	122.3 (13.1)	0.976
**Brachial DBP (mmHg)**	72.6 (7.1)	72.45 (7.6)	0.915
* **Central SBP (mmHg)**	115.6 (11.1)	116.7 (12.2)	0.660
* **Central DBP (mmHg)**	73.7 (7.1)	73.5 (7.7)	0.893
* **Central pulse pressure (mmHg)**	42.1(10.3)	43.3 (10.4)	0.581
* **Pulse wave velocity (m/s)**	8.0 (1.4)	7.8 (0.9)	0.426
* **Augmentation index (%)**	27.0 (7.2)	26.4 (6.5)	0.695

All values are means (standard deviations).

* Central blood pressures and stiffness measurements were obtained from 37 subjects in the placebo and 38 subjects in the vitamin D arms, respectively.

25OH Vitamin D = 25-hydroxyvitamin D.

DBP  =  diastolic blood pressure.

FMD  =  flow-mediated dilation.

HDL  =  high density lipoprotein.

LDL  =  low density lipoprotein.

SBP  =  systolic blood pressure.

**Figure 2:**
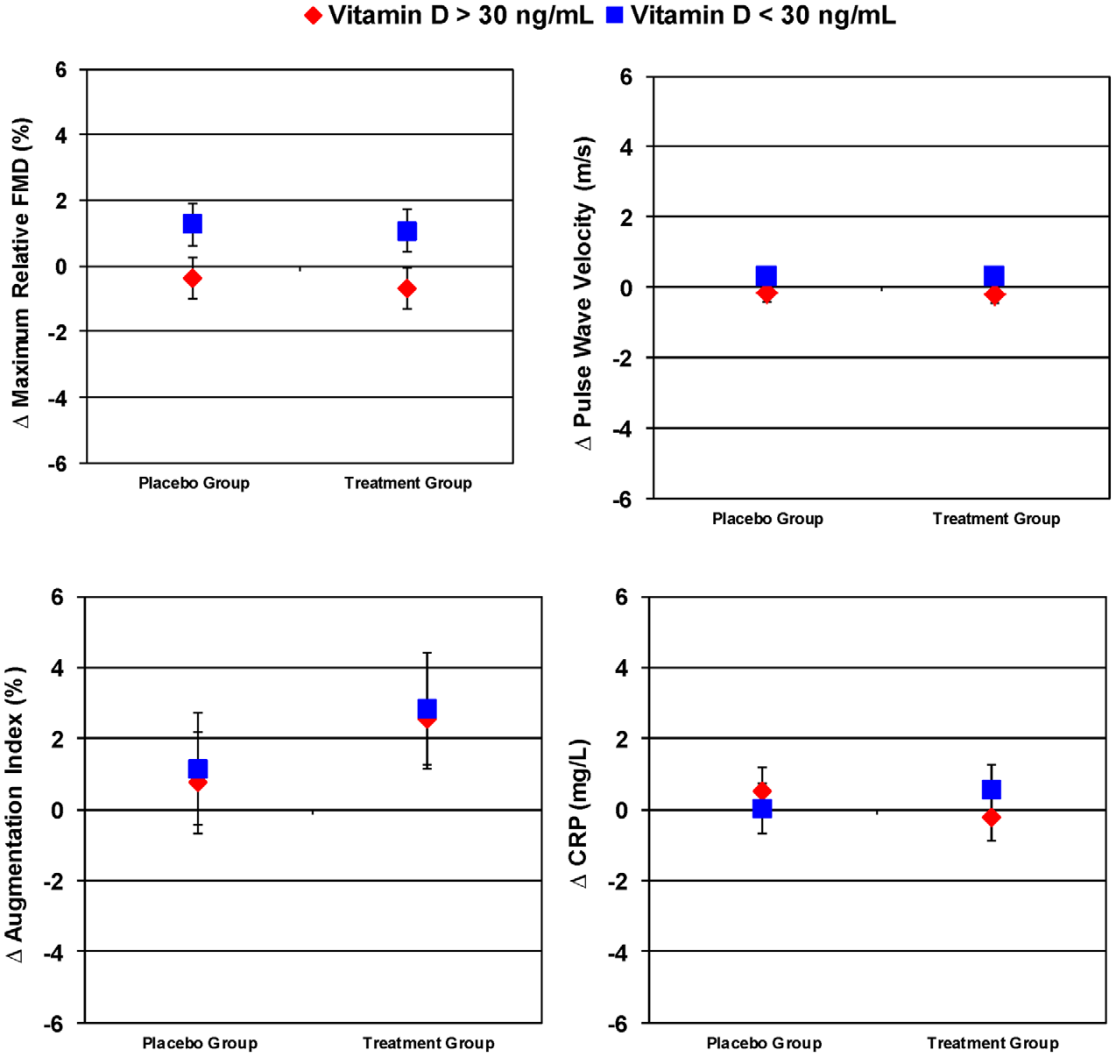
Interactions Between the Effects of Treatment Group and Baseline Vitamin D Status on Outcome Variables. Abbreviations as in Table 1.

**Table 2:** Changes from Baseline in Placebo vs. Treatment Groups after 4 Months.

Change	Placebo (N = 55)	Vitamin D (N = 55)	P-value between treatment groups
**25(OH) vitamin D (ng/mL)**	−0.2 (6.1)	15.7 (9.3)	<0.001
**Glucose (mg/dL)**	3.1 (10.4)	2.5 (10.2)	0.551
**Total/HDL cholesterol ratio**	0.2 (0.4)	0.1 (0.4)	<0.001
**C-reactive protein (mg/L)**	0.3 (1.9)	0.3 (4.1)	0.971
**Brachial artery diameter (cm)**	0.003 (0.009)	0.002 (0.012)	0.812
**Absolute FMD (cm)**	0.001 (0.009)	0.001 (0.013)	0.729
**Maximum relative FMD (%)**	0.27 (2.64)	0.33 (3.43)	0.767
**Heart rate (bpm)**	2.0 (5.5)	1.7 (5.4)	0.780
**Brachial SBP (mmHg)**	−2.5 (10.9)	−0.3 (8.4)	0.402
**Brachial DBP (mmHg)**	−0.4 (4.4)	−0.7 (5.1)	0.599
**Central SBP (mmHg)**	−2.1 (9.7)	−0.3 (7.0)	0.428
**Central DBP (mmHg)**	−0.5 (4.4)	−0.7 (5.1)	0.844
**Central pulse pressure (mmHg)**	−1.7 (8.9)	0.4 (8.6)	0.285
**Pulse Wave Velocity (m/s)**	0.00 (1.06)	0.05 (0.92)	0.652
**Augmentation index (%)**	0.9 (5.6)	2.7 (6.3)	0.096

All values are means (standard deviations).

* Central blood pressures and stiffness measurements were obtained from 37 subjects in the placebo and 38 subjects in the vitamin D arms, respectively.

Abbreviations as in Table 1.

### Ethics Statement

This study was approved by the University of Wisconsin Health Sciences Institutional Review Board. It was conducted according to the principles expressed in the Declaration of Helsinki. All subjects provided written consent.

### Study Design and Population

This was a prospective, randomized, double-blind, placebo-controlled trial of post-menopausal women with serum 25(OH)D concentrations between 10 and 60 ng/mL, as this range of vitamin D levels was thought to be representative of the majority of the US population, exclusive of those for whom withholding supplementation would not meet current standards of care. Participants were healthy, community-dwelling, ambulatory women from Madison, Wisconsin who were recruited between March 2009 and June 2010. Following informed consent, a screening evaluation of demographic and laboratory assessments were performed. Qualifying volunteers returned for a baseline visit. Endothelial function was evaluated by measuring brachial artery FMD in a core ultrasound laboratory using a standardized protocol described below [17], [22], [23]. Arterial stiffness was evaluated by determining carotid-to-femoral PWV and AIx using applanation tonometry, as described below [16], [24]. Laboratory evaluations were performed in a CLIA-approved lab and included fasting glucose, lipids, calcium, parathyroid hormone, and high-sensitivity C-reactive protein (CRP). Laboratory tests, FMD, and arterial stiffness studies were performed prior to initiating therapy and after 4 months. All measurements were performed in the morning after fasting for ≥8 hours. The first 34 subjects were enrolled as part of a pilot study that did not include arterial stiffness measurements. Arterial tonometry measurements were recorded in the remainder of the subjects after the techniques became available and recruitment was demonstrated to be feasible (Figure 1).

Exclusion criteria included: history of CVD, serum calcium >10.5 mg/dL, untreated primary hyperparathyroidism, history of nephrolithiasis, hypercalciuria, malignancy, tuberculosis, sarcoidosis, Paget's disease, malabsorption syndromes, estimated glomerular filtration rate ≤25 mL/minute, use of medications that interfere with vitamin D metabolism or affect bone turnover (including hormone replacement therapy), use of active metabolites of vitamin D within 6 months of screening, use of tanning beds or salons, or unwillingness to utilize sunscreen during periods of sun exposure of >15 minutes. All subjects agreed to avoid use of cod-liver oil and non-study vitamin D supplementation and to utilize sunscreen of SPF-15 or higher when sun exposure for >15 minutes.

### Randomization and Treatment Allocation

At the baseline study visit, volunteers were randomly assigned to receive either 2500 IU of oral D3 in a cookie or an identical tasting placebo cookie daily (D-Rich Foods, Inc., Manitowoc, Wisconsin). Randomization was carried out in a 1∶1 ratio without blocking using computer-generated random numbers. One individual in the Osteoporosis Research Center was assigned to randomize subjects and package vitamin D/placebo cookies with labels; this individual did not participate in recruitment, data collection, or analysis. All others including volunteers, study staff, investigators, and data analysts were blinded to the group assignment. There was no cross-over and all subjects were analyzed in an intention-to-treat manner. Enrollment was complete following recruitment of the pre-specified number of subjects.

### Outcomes

The primary outcome measures were brachial artery FMD, PWV, and AIx. Secondary outcomes included brachial and central aortic blood pressures, serum glucose, fasting lipid, and CRP levels. All tests were performed with subjects fasting between 7:00 and 11:00 AM.

### Measurement of Endothelial Function and Arterial Stiffness

Endothelial function was measured by ultrasound assessment of brachial artery FMD [17], [22], [23]. Subjects were placed in a supine position in a temperature-controlled room for 10 minutes before imaging. A blood pressure cuff was placed on the widest part of the proximal right forearm. Using an 8-MHz linear array vascular ultrasound transducer and a state-of-the-art ultrasound system (Acuson Sequoia 512, Siemens Medical Solutions, Issaquah, Washington), the brachial artery was located above the elbow and scanned longitudinally. After recording B-mode ultrasound images of the brachial artery and spectral Doppler velocities, the cuff was inflated to 250 mmHg for 5 minutes to induce reactive hyperemia. Immediately after deflation, spectral Doppler images were obtained to verify hyperemia. Brachial artery B-mode images were obtained 60 and 90 seconds after cuff release. Studies were recorded digitally; brachial artery diameters were measured in triplicate with a digital border tracing tool (Access Point Web 3.0, Freeland Systems, Westfield, Indiana). Studies were read in subject pairs (baseline and 4 months), blinded to study treatment. Reproducibility of measurements from this lab is excellent and has been reported recently using the exact same techniques, including during the time this study was being conducted [22], [23].

Arterial stiffness was measured by arterial tonometry (AtCor SphygmoCor Px, AtCor Medical, Sydney, Australia) [16], [24]. Tonometry recordings of the carotid and femoral arterial pulses were taken when a reproducible signal with a clear upstroke was obtained. The carotid-femoral PWV was determined by the intersecting tangents method [16], [24]. PWV (m/s) was calculated as the distance-to-transit time ratio of the recorded pulse wave. The time delay (seconds) from the electrocardiogram R-wave to the foot of the arterial pulse waveform was measured at the proximal (carotid) and distal (femoral) sites, based on an analysis of 10 seconds of stable tonometry tracings. The difference in the proximal and distal delay was considered to be the carotid-femoral transit time. The PWV distance was calculated as the difference in the absolute distance between the suprasternal notch and the carotid and femoral tonometry sites, respectively. AIx and central aortic pressures were derived from radial tonometry using a validated, generalized transfer function and calibrated using oscillometric brachial artery blood pressures. All AIx measurements were read independently and standardized to a heart rate of 75 bpm. An operator index greater than 85% was required for all analyzed tracings. Reproducibility of these measurements from this lab is excellent. For PWV, mean (standard deviation) differences for repeated measurements is 0.035 (0.44) m/s with an intra-class correlation coefficient of 0.91. For AIx and central pulse pressure, mean differences are 0.02 (4.0)% and 0.20 (2.8) mmHg, respectively, with intra-class correlation coefficients of 0.90 and 0.97, respectively.

### Laboratory Analyses

Fasting serum chemistry, glucose, lipids, and CRP levels and urinary calcium determinations were performed at General Medical Laboratories (Madison, WI). CRP was measured by nephelometry on a Siemens Vista analyzer using two control products to assess the daily performance of the instrument. The coefficient of variation was approximately 3.2%. Serum 25(OH)D was determined using reverse phase high-performance liquid chromatography as previously described [25], [26].

### Statistical Analysis and Sample Size

All analyses were conducted using STATA (Stata Statistical Software: Release 11, College Station, TX). Between groups comparisons were performed using two-tailed Student's t-tests or Mann-Whitney rank sum comparisons (for variables with non-normal distributions). Pearson correlations were used to determine associations with FMD. General linear models with 2 factors including group and vitamin D status (>30 vs <30 ng/mL), plus their interaction, were used to determine independent associations. All models were adjusted for body-mass index. Similar analyses were performed for PWV, AIx, central aortic pressures, CRP, and their changes. Bonferroni-adjusted p-values were reported for these analyses. A priori power calculations estimated that a minimum of 37 subjects in each arm would enable us to detect a 3% change in FMD with 80% power (alpha  =  0.05). The sample size was sufficient to detect an approximately 10% reduction in PWV with a standard deviation of approximately 15%.

## Results

### Subject Characteristics

Of 133 potential subjects that were screened, 114 were eligible (Figure 1). They were 63.9 (3.0) years old with a baseline 25(OH)D level of 31.3 (10.6) ng/mL (Table 1). The median baseline 25(OH)D level was 30.3 ng/mL. These subjects were randomized equally to each group; however, 2 subjects withdrew from each group and 1 subject in the placebo group did not have arterial stiffness measurements because of equipment failure. The final data analysis included 55 subjects in the treatment group and 54 in the placebo group. PWV and AIx were measured in 38 and 37 consecutive subjects in the treatment and placebo groups, respectively. Baseline characteristics were similar between groups with the exception of the treatment group having a slightly higher body-mass index (27.1 [4.7] vs. 25.3 [5.1] kg/m^2^, p = 0.022). There were no differences between groups in baseline brachial artery diameter (p = 0.66) or other measures of CVD risk including blood pressures, CRP, lipid profiles, or glucose level (all p>0.4) (Table 1).

### Effects of Treatment with VitD or Placebo

After 4 months, serum 25(OH)D increased by 15.7 (9.3) ng/mL in the treatment group versus −0.2 (6.1) ng/mL in those taking placebo (p<0.001). Despite improved VitD status, there were no significant differences between groups in any of the pre-specified outcomes including change in absolute FMD (0.001 [0.009] vs. 0.001 [0.013] cm p = 0.729), maximum relative FMD (0.3 [3.4] vs. 0.3 [2.6] %, p = 0.77), PWV (0.00 [1.06] vs. 0.05 [0.92] m/s, p = 0.65), AIx (2.7 [6.3] vs. 0.9 [5.6] %, p = 0.10), or CRP (0.3 [1.9] vs. 0.3 [4.2] mg/L, p = 0.97) (Table 2, Figure 2). Additionally, there were no significant changes in central aortic or brachial blood pressures (p>0.4). There was a small, statistically significant increase in the total/high-density lipoprotein cholesterol ratio in the placebo group compared to the treatment group (0.17 [0.36] vs. 0.07 [0.39], p<0.001). No adverse events were reported.

Of those in the treatment group, 92% (N = 51) had vitamin D levels >30 ng/mL after 4 months of therapy. General linear models showed no significant interaction between treatment group and VitD status for changes in maximum relative FMD (p = 0.65), PWV (p = 0.93), AIx (p = 0.97), or CRP (p = 0.26). No differences were observed after adjustment for baseline body-mass index.

Low VitD status (<30 ng/mL) was present in 47% (n = 54) of subjects at baseline. Subjects with low VitD status had a mean serum 25(OH)D level of 22.3 (standard deviation 5.26, range 9.1–29.0) ng/mL; lower than those with normal VitD status who had a mean serum 25(OH)D level of 39.7 (6.8, range 30.2–57.0) ng/mL. Low VitD status was associated with higher body-mass index (28.2 [5.0] vs. 24.2 [4.2] kg/m^2^, p<0.001), brachial systolic blood pressure (126.5 [13.3] vs. 118.6 [10.8] mmHg, p = 0.004), glucose (104.6 [42.3] vs. 90.8 [7.8] mg/dL, p = 0.003), CRP (2.9 [3.4] vs 1.5 [2.1] mg/L, p = 0.001), and lower maximum relative FMD (4.1 [3.0] vs. 5.4 [3.6] %, p = 0.043). After 4 months, those with low baseline 25(OH)D levels had a nominally statistically significant greater increase in maximum relative FMD than those with 25(OH)D levels >30 ng/mL (1.1 [2.4] vs.−0.5 [3.4]%, p = 0.002); however, this difference was independent of treatment group (Figure 2, Table S1).

## Discussion

We evaluated the effects of a higher dose of vitamin D supplementation (2500 IU daily) than used in the Women's Health Initiative on several vascular parameters and markers of CVD risk. In our study, treated subjects had circulating 25(OH)D levels that increased, on average, by over 15 ng/mL and over 90% of treated subjects achieved 25(OH)D levels >30 ng/mL. Nevertheless, we did not observe an improvement in FMD or arterial stiffness measures. Similarly, we did not observe differential improvements in central or peripheral blood pressures or CRP levels between treatment groups.

Our prospective, randomized, blinded study findings did not show improvements in FMD or either arterial stiffness measure after treatment with VitD. The few randomized controlled trials that have evaluated the effects of vitamin D supplementation on endothelial function or arterial stiffness have had mixed results in regard to FMD [19], [27], [28], PWV [29], and AIx [30]; however, those studies were notably smaller than our study and predominantly were performed in adults with medical conditions such as kidney disease and/or type II diabetes mellitus. Similarly, there are mixed reports regarding changes in blood pressure following VitD supplementation [15], [20]. Despite higher blood pressures in individuals with lower 25(OH)D levels, we found no change in blood pressure after VitD supplementation, in agreement with the largest of these trials [20]. Despite higher CRP levels in individuals with lower 25(OH)D levels, we also found no change in CRP after supplementation, a finding consistent with previous reports [30]–[32]. The lack of an effect of VitD supplementation on these markers of CVD risk was confirmed in our subgroup analyses restricted to participants with low VitD status at baseline using multivariable linear models which showed that treatment group did not influence change in FMD, PWV, or AIx. Although individuals with low VitD status at baseline had a small increase in maximum relative FMD after 4 months, this increase was not influenced by treatment group. Our results challenge the hypothesis that VitD supplementation reduces CVD risk.

Vitamin D receptors are widely distributed throughout the body and have been isolated from vascular endothelial cells and cardiac myocytes [8]. Mechanistically it is conceivable that low VitD levels could have deleterious CVD effects by dysregulation of systemic calcium metabolism, as cardiac myocyte contraction depends on calcium homeostasis and since coronary artery calcification is predictive of CVD risk [6], [8], [9]. Also, VitD modulates lymphocytic cytokine production [33], potentially affecting growth and proliferation of vascular smooth muscle cells and cardiomyocytes [34], stimulating vascular tissue anticoagulant activity [35], and suppressing renin gene expression [36], which could lead to the clinical manifestations of hypertension, coronary artery disease, and congestive heart failure. However, mechanistic hypotheses do not necessarily imply that supplementing individuals with low 25(OH)D levels will reverse these adverse processes and reduce CVD risk [37]. Blood pressure reductions have been observed with VitD supplementation [11], [38], but the majority of studies have shown no change [15], [20], [28]. Furthermore, 2 randomized controlled trials showed no improvement in left ventricular function in patients with heart failure randomized to VitD therapy [39], [40].

Although plausible biological mechanisms and epidemiological data suggest that VitD deficiency may increase and VitD supplementation may reduce CVD risk, observational studies cannot account for unmeasured confounders. In our study, as in others, individuals with low VitD tended to have a greater CVD risk factor burden [5], [8], [10]. It has been proposed that low VitD is responsible for these observations; however, it is possible that these CVD risk factors simply are associated with lower 25(OH)D levels. Indeed, previous studies have shown that the risk factors for VitD deficiency are similar to traditional CVD risk factors [8]. In our study, despite an average increase in circulating 25(OH)D levels of nearly 16 ng/mL, we observed no improvements in FMD, PWV or AIx, surrogate CVD risk markers that predict initial and recurrent CVD events [14].

### Limitations

Our endpoints are surrogate markers for CVD risk; we did not evaluate CVD death, myocardial infarction, or stroke. Although our markers are predictive of CVD events and are well-established research tools, they are imperfect and an absence of change in these measures does not exclude the possibility that VitD supplementation may reduce CVD risk [14], [17], [41]. Participants in this study were generally healthy post-menopausal women with typical 25(OH)D levels. It is possible that VitD supplementation in men or in individuals with higher baseline CVD risk or certain co-morbidities, including abnormal endothelial function or lower 25(OH)D levels, may reduce CVD risk. Additionally, research on the effects of VitD supplementation is challenged by the absence of a widely accepted definition of vitamin D “insufficiency.” Proposed “reasonable” 25(OH)D levels range from 20 ng/mL (50 nmol/L) to 32 ng/mL (80 nmol/L) [42], [43] and some advocate VitD supplementation in individuals with 25(OH)D levels that are <50 ng/mL [44]. Subjects in this study started with a wide range of 25(OH)D levels; those subjects with higher starting levels may have had a blunted arterial response to treatment despite potentially being randomized to the treatment group. Similarly, there is considerable debate regarding the optimal dose for VitD supplementation and repletion in adults. Expert opinion for supplemental dosing ranges from 600–1000 IU (20–25 µg) to 2000 IU (50 µg) daily [43], [45], [46], although some have argued that even the latter dose may be inadequate and higher doses frequently are used clinically [42]. Given the inconsistencies in the literature, we cannot exclude the possibility that supplementation with even higher doses of VitD for a longer duration would show a benefit in terms of CVD risk; however, interventions that reduce CVD risk also tend to improve endothelial function and arterial stiffness soon after initiating treatment, suggesting that 4 months was a reasonable duration of treatment [14], [17], [41]. Based on revised estimates using a mixed effects general linear model and restricted maximum likelihood estimation, with 55 subjects per arm we had over 90% power to detect a 1.5% difference in FMD. For PWV, we observed essentially no change PWV and the SDs were very small, however there still is a small chance that we missed a true difference between groups.

### Conclusions

In the largest prospective randomized clinical trial of VitD supplementation that used a dose of VitD that normalized VitD levels in most participants, VitD supplementation did not improve endothelial function, arterial stiffness, reduce CRP or improve blood pressure in healthy, post-menopausal women. This study does not support the use of VitD supplementation to reduce CVD risk; however, long-term outcomes studies of this intervention are needed.

## Supporting Information

Checklist S1 **CONSORT Checklist.** CONSORT 2010 checklist of information to include when reporting a randomized trial.
(DOC)

Protocol S1 **Trial Protocol.** Effect of Vitamin D Status on Endothelial Function.
(DOC)

Table S1 Unadjusted Changes from Baseline after 4 Months Stratified by Baseline Vitamin D Level.
(DOCX)
