# Supplementary material for: A Prospective Randomized Controlled Trial of the Effects of Vitamin D Supplementation on Cardiovascular Disease Risk
Source: PLoS One. 2012 May 7;7(5):e36617. doi: 10.1371/journal.pone.0036617 (PMC3346736; doi:10.1371/journal.pone.0036617)
Supplement: Protocol S1 — Trial Protocol. Effect of Vitamin D Status on Endothelial Function. (DOC) [file pone.0036617.s002.doc]

**Title:**

**Effect of Vitamin D Status on Endothelial Function**

**Investigators:**

Rekha Ramamurthy, M.D.

Neil Binkley, M.D.

James Stein, M.D.

**Site:**

University of Wisconsin Osteoporosis Research Program

2870 University Avenue, Suite 100

Madison Wisconsin 53705

608.265.6410

**IRB:**

University of Wisconsin Health Sciences Human Subjects Committee

**Version 2: September 30, 2008**

**Table of Contents:**

| Protocol Overview and Introduction ……………………………………... | 3 |
| --- | --- |
| Background ………………………………………………………………… | 3-5 |
| Hypotheses and Specific Aims ……………………………………..……. | 5 |
| Study Design …………………………………………...………. | 5 |
| Inclusion/Exclusion Criteria ………………………………………………. | 6 |
| Study Event Flow Chart …………………………………………………... | 7 |
| Food Supplement …………………………………………………………. | 7 |
| Laboratory Analyses ………………………………………………………. | 7 |
| Safety ……………………………………………………………………….. | 7-8 |
| Data Handling ……………………………………………………………… | 8 |
| Power Calculation …………………………………………………………. | 8 |
| Statistical Analyses ……………………………….………………………. | 8 |
| Timeline …….………………………………………………………………. | 8 |
| References .………………………………………………………………. | 8-12 |

**PROTOCOL OVERVIEW AND INTRODUCTION:**

In the United States, cardiovascular disease causes over one-third of all deaths and vitamin D deficiency is epidemic. An increasing body of data suggests that low vitamin D status adversely impacts the cardiovascular system. It is our fundamental hypothesis that vitamin D deficiency is a risk factor for cardiovascular disease by causing a cytokine mediated pro-inflammatory milieu leading to endothelial dysfunction. Moreover, we hypothesize that vitamin D supplementation will reduce inflammation, thus restoring endothelial function and thereby reducing cardiovascular disease risk. To begin testing these hypotheses, the specific aims of this pilot study are to investigate the effect of vitamin D baseline status (as defined by serum 25-hydroxyvitamin D [25(OH)D] measurement) and subsequent vitamin D therapy on endothelial function, arterial stiffness, and selected plasma biomarkers of inflammation.

This pilot research will be conducted in 80 post-menopausal women where 40 will receive vitamin D_3_ 2,500 IU daily, the others matching placebo. This study will explore the effects of vitamin D on plasma markers of inflammation, endothelial function, and arterial stiffness. Postmenopausal women aged 55-65 years are chosen due to their highest risk for development of a subsequent new cardiovascular disease diagnosis. All study participants will have fasting laboratory and noninvasive vascular ultrasound studies performed at baseline and four months later. The primary outcome measure of this pilot study is change in markers of endothelial function and arterial stiffness with vitamin D_3_ therapy. Changes in plasma biomarkers of inflammation with vitamin D_3_ therapy are secondary outcome measures. If these hypotheses are correct, our long-term goals include investigation of the effect of vitamin D repletion on subclinical atherosclerosis and subsequent cardiovascular events.

**BACKGROUND**

**A. Cardiovascular Disease**

Cardiovascular disease (CVD) is the leading cause of mortality worldwide and causes over 36% of deaths annually in the United States. There is general agreement that age, family history, smoking status, dyslipidemia, hypertension and diabetes mellitus are causally linked risk factors for CVD. However, currently unappreciated CV risk factors may well exist; one such factor could be vitamin D inadequacy.(1)

**B. Vitamin D**

The vitamin D status of an individual is best evaluated by measurement of circulating 25(OH)D concentration. The optimal circulating 25(OH)D level has been suggested to be ~30 ng/ml or above, a value associated with maximal parathyroid hormone suppression and reduced fracture rate.(2) Using this definition, it has been estimated that approximately half of all adults in the United States have inadequate vitamin D status.(3) Therefore, if low vitamin D status contributes to the development of CVD, it is plausible that a large portion of the population may potentially be targeted for risk modification/reduction by optimization of their vitamin D status.

Vitamin D is produced in the skin when 7-dehydrocholesterol is converted to vitamin D_3_ (cholecalciferol) by ultraviolet B radiation.(4) Additionally, dietary or supplement intake of vitamin D contributes to circulating levels of vitamin D_2_ (ergocalciferol) and D_3_. Subsequently, vitamin D is activated by hydroxylation at 25 and 1 carbons in the liver and kidney respectively to form the active form, 1,25-dihydroxyvitamin D (calcitriol).(5) Calcitriol is important in intestinal calcium transport; thus, vitamin D deficiency has classically been recognized to cause decreased calcium absorption and impaired bone mineralization. However, it is increasingly being recognized that both 25-hydroxyvitamin D and 1,25-dihydroxyvitamin D are important in optimal function of multiple tissues and organ systems.(6) The CV system may be one such system.

**C. Vitamin D and the Cardiovascular System**

Although it has been classically accepted that 1-α-hydroxylation of 25-hydroxyvitamin D [25(OH)D] to the active form, 1,25-dihydroxvitamin D [1-25(OH)_2_D] occurs in the kidney, it is now appreciated that 1-25(OH)_2_D is also produced by local 1α-hydroxylases in various extra-renal tissues. Extra-renal 1-25(OH)_2_D production is dependent on circulating levels of 25(OH)D. As locally produced 1-25(OH)_2_D plays an important autocrine role,(6) vitamin D deficiency could be expected to have adverse local consequences. For example, in the CV system, as vitamin D receptors are present in endothelium,(7) vascular smooth muscle(8, 9) and cardiomyocytes,(10) it is possible that low vitamin D status could adversely affect both cardiac and vascular function. Moreover, circulating 1-25(OH)_2_D_3_ is an important regulator of systemic calcium metabolism and intracellular calcium homeostasis in various tissues.(6) Altered calcium dynamics due to vitamin D deficiency in smooth and/or cardiac myocytes could plausibly adversely affect CV function. Additional non-classical vitamin D actions include modulation of lymphocytic cytokine production,(11-13) regulation of growth and proliferation of vascular smooth muscle and cardiomyocytes,(14) stimulation of vascular tissue anticoagulant activity(15) and suppression of renin gene expression.(16-18). Based on the above, it is clear that vitamin D deficiency could plausibly contribute to increased CV risk.

In fact, CVD prevalence is related to availability of ultraviolet solar exposure in that epidemiologic data finds coronary artery disease (CAD) and hypertension rates to increase with distance from the equator. Specifically, greater CVD prevalence is observed in regions with less exposure to sunlight.(19-22) Cross-sectional studies have reported associations between lower vitamin D levels and plasma renin activity,(23) blood pressure(24, 25), coronary artery calcification(26, 27) and CVD (28, 29). A recent longitudinal prospective study involving offspring of the Framingham cohort supported earlier findings associating vitamin D deficiency with incident CVD.(30) In summary, biologically plausible mechanisms combined with epidemiological data suggest that vitamin D deficiency may be an unappreciated risk factor for the development of CVD. Despite this association of vitamin D deficiency and risk of CVD, the underlying mechanism(s) remain unknown. Altered endothelial function may be the key link for this association.

**D. Endothelial Dysfunction, Cardiovascular Disease and Vitamin D Status**

Measurement of endothelial function has recently emerged as a useful CVD research tool and serves as a “barometer” for CV health.(31) For example, severity of endothelial dysfunction relates to the risk for initial or recurrent CV event.(32-35) Additionally, a number of interventions that reduce CV risk also improve endothelial function.(36-41) This could be anticipated in that the endothelium acts to maintain vascular homeostasis through multiple complex interactions with cells in the vessel wall and lumen.(42) The endothelium regulates vascular tone by balancing production of vasodilators and vasoconstrictors. It modulates blood fluidity and coagulation through production of factors that regulate platelet activity, as well as the clotting cascade and fibrinolytic system. Finally, the endothelium has the capacity to produce cytokines and adhesion molecules that regulate and direct the inflammatory process.(43) In healthy individuals, the endothelium responds to these stimuli by releasing vasodilatory factors, particularly nitric oxide [NO]. People with angiographically proven CAD display impaired flow-mediated dilation [FMD] reflecting loss of NO and unopposed constrictor effects.(44) Measures of arterial stiffness that are affected by endothelial function, including pulse wave velocity, central aortic blood pressure and augmentation index, predict CV events.(45, 46) As such, the endpoints chosen for this research (FMD, pulse wave velocity, central aortic pressure, and aortic augmentation index) are accepted surrogates of endothelial function and arterial stiffness.

In a healthy condition, the endothelium maintains normal vascular tone and blood fluidity. There is little to no expression of pro-inflammatory cytokines. However, traditional and novel CVD risk factors initiate a chronic inflammatory process that is accompanied by a loss of vasodilator and anti-thrombotic factors and an increase in vasoconstrictor and pro-thrombotic products. In this regard, vitamin D also has intriguing immunoregulatory properties.(47, 48) For example, reduced levels of C-reactive protein (CRP), erythrocyte sedimentation rate and cytokines have been noted with higher 25(OH)D levels or after vitamin D supplementation in patients with multiple sclerosis(49, 50) and ankylosing spondylitis(51). Consistent with this, vitamin D may be of therapeutic benefit in several autoimmune disease and allograft rejection(52, 53) and may modulate disease severity by reducing inflammation. In two small trials, vitamin D supplementation lowered CRP levels.(54, 55) Reduction in inflammatory cytokines was also noted in patients with congestive heart failure when supplemented with vitamin D.(56, 57) This is intriguing given the well-documented association of CRP with CV risk as either a marker of inflammation or possibly even in a causal role.(58, 59) However, the possibility that vitamin D deficiency is causally related to CVD development via enhanced cytokine production remains unstudied.

Statins, agents accepted to reduce CV risk, may have anti-inflammatory and endothelial protective effects in addition to reducing lipids. It is plausible that some of these beneficial effects are related to vitamin D as a recent study found atorvastatin to raise serum 25(OH)D levels among patients with a recent acute coronary syndrome event.(60) It has been hypothesized that statins may even activate vitamin D receptors.(61) Vitamin D levels may have beneficial effects on the vascular system by reducing arterial stiffness. In a cohort of patients with end-stage renal disease, both serum 25(OH)D and 1-25(OH)_2_D were negatively correlated with aortic pulse wave velocity and positively correlated with brachial artery distensibility and FMD after adjustment for blood pressure and age.(62) Thus it is possible that the beneficial CV effects of statins are, in part, associated with vitamin D.

**E. Vitamin D, cardiovascular disease and the immune system**

It is probable that vitamin D has important immunomodulatory effects. Identification of vitamin D receptors (VDRs) in peripheral blood mononuclear cells sparked an early interest in vitamin D as an immune regulator.(63-65) Helper T (Th) cells are central to all antigen-specific immune responses. Th-1 cells secrete multiple pro-inflammatory cytokines including tumor necrosis factor (TNF), which are essential for cell-mediated immune responses. Th-2 cells play an important role in antibody-mediated immunity. The balance of Th cell responses dictates the outcome of any given challenge to the immune system. Both Th-1 and Th-2 cells are known targets of 1-25(OH)_2_D_3_. Vitamin D causes a relative shift away from Th-1 profile and toward a Th-2 like profile.(66) The end result of these changes is a switch away from a pro-inflammatory cytokine profile to a more anti-inflammatory profile.(67-70) Given this, it is not surprising that vitamin D deficiency has been associated with an increased risk of Th-1 cytokine-mediated autoimmune diseases such as inflammatory bowel disease, rheumatoid arthritis, systemic lupus erythematosis, multiple sclerosis, and type 1 diabetes mellitus.(71-73)

Inflammation is thought to be a major process mediating accelerated progression of atherosclerosis and its complications.(74) Manifold pathways tightly link inflammation with early development of atherosclerosis and plaque rupture.(75-77) Various inflammatory factors including interleukin 6 (IL-6), TNF, and CRP are considered to be actively involved in atherogenesis and contribute to plaque instability, thrombosis and acute coronary syndromes.(78-80) Activation of endothelial cells leads to expression of various cell adhesion molecules (CAMs) that are responsible for mediating cell-cell interaction between immune system and the endothelium. Upregulation of CAMs is a dynamic process sensitive to inflammatory cytokines. Levels of certain CAMs (ICAM-1 and VCAM-1) are thought to be indicators of inflammation and early atherosclerosis.(81-83) CRP has been shown to predict CV risk, independent of traditional predictive factors.(84-86) IL-6 levels are increased both in stable CAD and acute coronary syndromes,(75) and cardiomyopathies.(87) Several studies evaluated the predictive CVD value of IL-6 in healthy individuals independent of other inflammatory markers.(88) This may also be true in those with established CAD.(89) TNF-α also seems to have predictive value for CV outcome in adults with (90) and without underlying CVD.(91) In the ABC study,(87) a composite summary indicator of inflammation, including CRP, TNF-α and IL-6, showed a strong association with incident CVD and congestive heart failure in patients without CVD. Those with elevation of all three indicators in the highest tertile had a 2-3 fold higher risk.

Vitamin D treatment has been shown to inhibit production of ICAM-1,(92-94) VCAM-1 (93, 95) and cytokine production (56, 70, 96) both *in vitro* and *in vivo* settings. In summary, it is plausible that vitamin D deficiency leads to increases in pro-inflammatory cytokines, thereby contributing to the development and progression of CVD. This initial pilot work will begin to explore this possibility by evaluating the effect of vitamin D status on both endothelial function, arterial stiffness and plasma biomarkers of inflammation in postmenopausal women prior to, and following four months of treatment with vitamin D.

**HYPOTHESES AND SPECIFIC AIMS**

Our fundamental hypothesis is that low vitamin D status is a risk factor for CVD by causing a proinflammatory milieu, thereby leading to endothelial dysfunction. Additionally, we hypothesize that vitamin D supplementation will reduce inflammation, thereby restoring endothelial function and ultimately reducing CVD risk. To begin testing these hypotheses, the specific aims of this pilot study are to investigate:

1. The effect of vitamin D status, as defined by serum 25-hydroxyvitamin D [25(OH)D], on endothelial function, arterial stiffness, and selected plasma biomarkers of inflammation.
2. The effect of subsequent vitamin D therapy on endothelial function, arterial stiffness, and selected plasma biomarkers of inflammation.

To this end, we hypothesize that low vitamin D status is associated with increased circulating levels of inflammatory biomarkers. Though the primary outcome measure of this pilot study is change in markers of endothelial function and arterial stiffness with vitamin D_3_ therapy, changes in circulating inflammatory biomarkers are being proposed here as secondary outcome variables. If these hypotheses are correct, our long-term goals include investigation of the effect of vitamin D repletion on subclinical atherosclerosis and subsequent cardiovascular events. Ultimately, these future studies will be necessary to determine whether vitamin D supplementation could reduce future CVD events and mortality.

**STUDY DESIGN AND METHODS**

**Specific Aim #1. To investigate the effect of vitamin D status, as defined by serum 25-hydroxyvitamin D measurement, on endothelial function, arterial stiffness and plasma biomarkers of inflammation.** This study will utilize total of 80 postmenopausal women. At the screening visit, following informed consent, a baseline evaluation consisting of basic demographic information, including age, ethnicity, height, and weight will be collected. Clinical information will include personal and family medical history as well as medication and supplement use. Screening laboratory studies consisting of serum chemistry panel, 25(OH)D, and 24-hour urine calcium will be obtained. Subsequently, qualifying volunteers will return for a baseline visit at which time a complete physical examination will be performed, including blood pressure and pulse measurements. Laboratory data will be obtained, which will include fasting glucose, fasting lipid profile, plasma inflammatory biomarkers (i.e. hsCRP, IL-6, TNF-α, ICAM-1 and VCAM-1), serum calcium, bone turnover markers and parathyroid hormone. The potential impact of vitamin D on endothelial function and arterial stiffness, which are surrogate markers for CVD, will be explored using noninvasive ultrasound technology and tonometry at baseline and again after four months.

**Specific Aim #2. To investigate the effect of vitamin D supplementation on endothelial function, arterial stiffness and plasma biomarkers of inflammation.** At the time of their baseline study visit, volunteers will be randomly assigned to receive either 2,500 IU D_3_ daily or placebo. Both volunteers and study staff will be blinded to the group assignment. Subsequent study visits will occur after one, two, and four months. Participants will return to the Osteoporosis Research Center in a fasting state for blood draw, which will be done between 0700 and 1100. Serum 25(OH)D and serum calcium will be measured at all of the time points. Fasting plasma glucose, fasting lipid profile, plasma biomarkers of inflammation, bone turnover markers and PTH along with brachial artery FMD and tonometry measurements will be repeated at the four-month study conclusion visit.


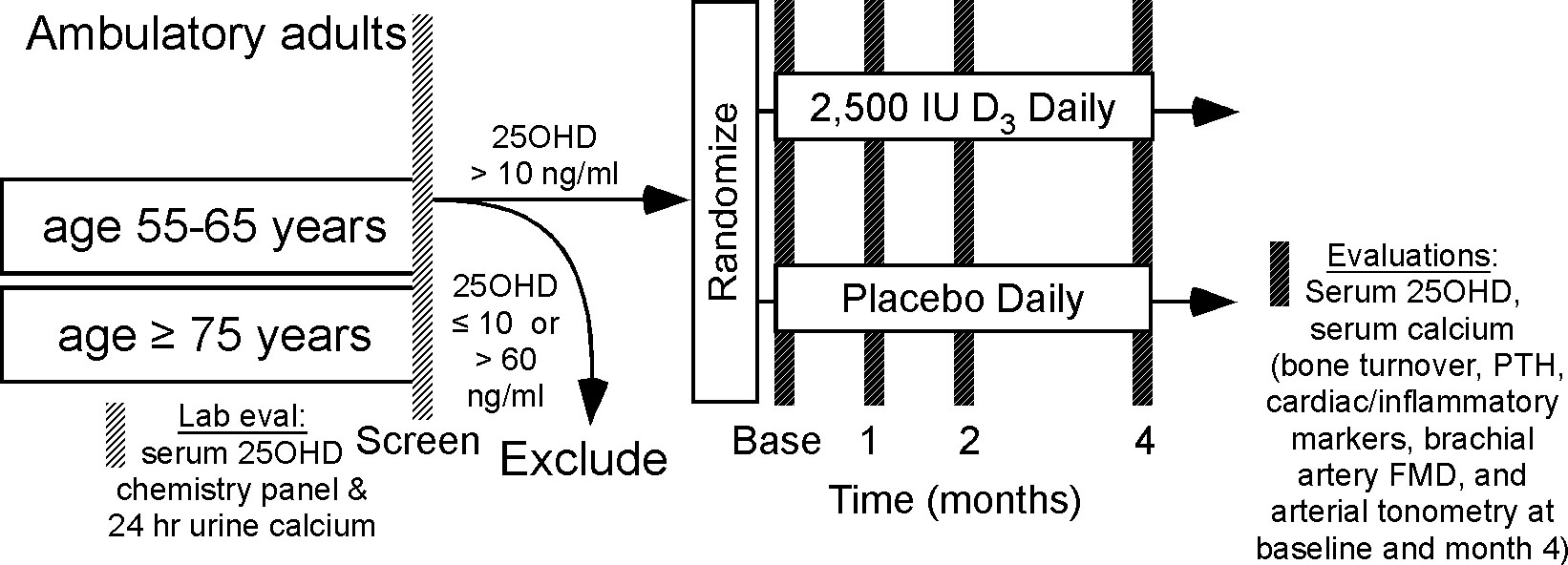


Inclusion criteria:

- Healthy, community-dwelling ambulatory post-menopausal women.
- Able and willing to sign informed consent.
- Ages: 55-65.
- Baseline serum 25OHD concentration > 10 ng/ml and < 60 ng/ml
- Not pregnant
- Willing to avoid use of cod-liver oil and non-study vitamin D supplementation; standard multiple vitamins containing ≤ 400 IU used no more than once daily will be allowed.
- Willing to utilize sunscreen of SPF-15 or higher when sun exposure for more than 15 minutes is expected.
- Willing to fast for 12 hours.

Exclusion criteria:

- Current hypercalcemia (serum calcium > 10.5 mg/dl) or untreated primary hyperparathyroidism.
- History of nephrolithiasis
- Baseline 24-hour urine calcium > 250 mg
- Known risk factors for hypercalcemia, e.g., malignancy, tuberculosis, sarcoidosis, Paget’s disease.
- History of any form of cancer within the past five years with the exception of adequately treated squamous cell or basal cell skin carcinoma.
- Known previous personal history of cardiovascular disease.
- Renal failure; defined as a calculated creatinine clearance (using the Cockroft-Gault approach) of ≤ 25 ml/minute.
- Severe end-organ disease, e.g., cardiovascular, hepatic, hematologic, pulmonary, etc., which might limit the ability to complete this study.
- Treatment with any drug known to interfere with vitamin D metabolism, e.g., phenytoin, phenobarbital.
- Known malabsorption syndromes, e.g., celiac disease, active inflammatory bowel disease, etc.
- Known allergy to chocolate.
- Use of medications known to alter bone turnover including bisphosphonates, estrogen, selective estrogen receptor modulators, parathyroid hormone, testosterone or calcitonin.
- Treatment with any active metabolites of vitamin D, e.g., calcitriol, within six months of screening.
- Use of tanning beds or salons or unwillingness to utilize sunscreen during periods of sun exposure of 15 minutes or longer.

All participants will be reimbursed $20 for each study visit. Project personnel will not receive recruiting incentives.

**STUDY VISIT EVENT FLOW CHART**

| **Time (months)** | **-1** | **0** | **1** | **2** | **4** |
| --- | --- | --- | --- | --- | --- |
| Study visit | Screen | Base | 1 | 2 | 3 |
| Consent | X |  |  |  |  |
| Medical history | X |  |  |  |  |
| Physical examination |  | X |  |  |  |
| Flow-Mediated Vasodilation |  | X |  |  | X |
| Radial Tonometry |  | X |  |  | X |
| Serum chemistry | X |  |  |  | X |
| Serum calcium |  | X | X | X |  |
| 24-hour urine calcium | X |  |  |  |  |
| 25(OH)D | X | X | X | X | X |
| PTH |  | X |  |  | X |
| Bone turnover markers |  | X |  |  | X |
| Fasting glucose |  | X |  |  | X |
| Fasting lipid panel |  | X |  |  | X |
| C-reactive protein (hsCRP) |  | X |  |  | X |
| Inflammatory Biomarkers |  | X |  |  | X |
| Dispense supplement |  | X | X | X |  |
| Assess compliance |  |  | X | X | X |
| Adverse event recording |  | X | X | X | X |

**Study Food Supplement:**

In this randomized trial, the study preparation will be a low calorie (~65 calories) cookie-like disc containing 2,500 IU of vitamin D_3_ or placebo produced by D-Rich Foods, Inc., Manitowoc, Wisconsin. Prior to study initiation the vitamin D content of the study preparation will be independently validated in the laboratory of Dr. H. DeLuca at the University of Wisconsin. Study participants will be asked to store the study supplement at room temperature. All unused study supplement will be returned and counted to document compliance.

**Study Randomization:**

An individual in the UW-Osteoporosis Research Center will be assigned to randomize subjects and package vitamin D/placebo “cookies” with labels. This individual will not participate in recruitment or data collection. We will ensure that the staff will remain blinded. The PRC has determined that this does not require their participation as this is not a drug.

**Laboratory Analyses:**

Serum chemistry, fasting glucose, fasting lipid panel, high sensitivity C-reactive protein (hsCRP), and urinary calcium determinations will be performed at General Medical Laboratories (Madison, WI) in routine clinical manner. Serum 25(OH)D concentration will be determined using reverse phase HPLC in the laboratory of Dr. Marc Drezner at the William S. Middleton VAMC (Madison, WI). Serum intact PTH and bone turnover markers will be run in Dr. Binkley’s laboratory using commercially available kits. Plasma inflammatory biomarkers will be run at the Wisconsin Regional Primate Research Center Laboratory using commercially available assay kits.

**Safety:**

The risks of participation in this study are extremely low. Specific risks due to venipuncture include pain, bruising and infection.

Ultrasonography is used in FMD measurement. Arterial tonometry is used in pulse wave velocity, central aortic pressure and aortic augmentation index determination. These are low risk imaging procedures and are commonly used noninvasive techniques in the field of cardiovascular research to evaluate for endothelial dysfunction and arterial stiffness, which are considered surrogate markers for cardiovascular disease. No significant adverse outcomes have been reported from the use of these techniques.

Safety concerns regarding the potential development of D toxicity manifesting as hypercalcemia and/or hypercalcuria could potentially be raised. However, the risk of D toxicity with the doses utilized in this study is extremely remote. All high quality reports of D toxicity involve the long-term intake of > 40,000 IU daily. As the maximum vitamin D dose administered over the four months of this trial will be 300,000 IU, this study has a wide margin of safety. Based upon published literature and our clinical experience, we believe the risk of vitamin D toxicity from study supplementation to be negligible. Given this, we do not believe that a formal data and safety monitoring board is necessary.

Despite the wide safety margin noted above, we have taken additional steps to assure participant safety by use of study criteria selected to exclude those with existing hypercalcuria, conditions associated with increased likelihood of vitamin D toxicity, e.g., tuberculosis, and the rare individual with high vitamin D status. Adverse events will be collected and recorded on case report forms for completeness and reported at time of study publication.

An additional potential risk of study participation is breach of confidentiality. To minimize this risk, all data will be coded with subject’s initials and ID number. This is to ensure confidentiality. Moreover, all information collected in this research study will be stored on password-protected computers or in locked cabinets at the University of Wisconsin-Madison Osteoporosis Clinical Research Program.

**Data Handling and Analysis**

A study chart will be prepared for all participants and every study encounter will be recorded on case report forms (CRF’s). Hard copies of CRF’s and laboratory reports will be kept in each participant’s study chart, which will be stored in the study coordinator’s office at 2870 University Avenue. The study chart storage cabinet and the study coordinators office are both locked after working hours.

All study data will be entered into an Excel database by the study coordinator or designee. This database will reside on UW Osteoporosis Clinical Research server located at 2870 University Ave. All OCRC computers are password protected and backed up daily utilizing a tape drive system. To assure that the data are transmitted reliably from paper into electronic form, two different individuals, or one individual on different days, will enter all data. Excel will verify that identical values were entered. Any discrepancies will be corrected by referring to the CRFs. At SMDC, all data will be entered into an access database. As at UW, dual-entry of data will be performed on 100% of data.

**ANALYTICAL APPROACHES AND CONSIDERATIONS**

1. **Power Calculation**

In experienced laboratories with excellent reproducibility (such as in Dr. Stein’s CV research lab), a 2% to 3% improvement in FMD can be detected in parallel-groups trials with about 25 to 45 subjects per treatment arm.(97-99) Power calculation shows that 37 subjects in each arm of this parallel group study should enable us to detect a 3% change in FMD with 80% power using a two-sample t-test with two-sided 5% level if the standard deviation of %FMD is similar to that observed by Sorenson.(98) Allowing for an 8% drop out rate, we plan to recruit 40 subjects per arm.

Endothelial function will be evaluated by measuring brachial artery FMD using ultrasound. Studies will be read in subject pairs (baseline and four months), blinded to study time point and treatment. Output data will include heart rate, brachial artery blood pressure, brachial artery diameter, reactive hyperemia VTI, and FMD at each time point.

Arterial stiffness will be determined from arterial tonometry using an AtCor Sphygmacor Px arterial tonometry system. Studies also will be read as described above. Output data will include heart rate, derived pulse wave velocity, central aortic systolic and diastolic pressure and aortic augmentation index, read independently and standardized to a heart rate of 75 bpm. The proposed sample size (37 per group or 74 total) will be sufficient to detect an ~10% reduction in pulse wave velocity with a standard deviation of approximately 15%, with at least 80% power using a two-sample, two-sided t-test with 5% level of significance. Thus, allowing for an 8% drop out rate, we plan to recruit 40 subjects per arm. Extrapolating from population-based studies,(100, 101) a 10% change in pulse wave velocity would predict a significant reduction in CVD risk.

1. **Statistical Analysis**

The primary endpoint is change in endothelial function from baseline after 16 weeks of vitamin D supplementation compared to placebo. The primary comparison will be made using a two-tailed Student's t-test. Multivariable linear regression modeling will be used to evaluate predictors of changes in FMD, including 25(OH)D levels, age, sex, and other CV risk factors. For the cross-sectional baseline analysis, Pearson correlations will be used to determine associations with FMD, and multivariable linear regression modeling will be used to determine independent baseline associations. Similar analyses will be performed for pulse wave velocity, central aortic pressure and aortic augmentation index.

Similar analyses will be performed for changes in plasma biomarker levels (hs-CRP, IL-6, TNF-α, sICAM and sVCAM) from baseline to the 16-week follow-up. The proposed sample size of 37 subjects per group will permit us to detect differences between groups of 0.6-0.7 pg/ml in IL-6, 0.4-0.5 pg/ml in TNF-α and 39-53 ng/ml in sICAM levels using a two-sample t-test with Type 1 error rate of 5% and 80% power if the standard deviations of cytokine levels are similar to those observed by Marketou.(102) As stated above, we plan to recruit 40 subjects per arm allowing for an 8% drop out rate.

**STUDY TIMELINE**

34 subjects were recruited as part of protocol 2007-0211 for initial pilot data. 80 additional subjects will need to be recruited as part of this study. Additionally, it will be necessary to obtain vitamin D and placebo chocolate discs, which will require analysis. We anticipate to have validated study preparation on site by the Fall of 2008. Furthermore, we have allowed up to 6 months for recruitment, making our estimated end date August 2009.

**REFERENCES**

1. **Zittermann A, Schleithoff SS, Koerfer R** 2005 Putting cardiovascular disease and vitamin D insufficiency into perspective. Br J Nutr 94:483-492

2. **Dawson-Hughes B, Heaney RP, Holick MF, Lips P, Meunier PJ, Vieth R** 2005 Estimates of optimal vitamin D status. Osteoporos Int 16:713-716

3. **Zadshir A, Tareen N, Pan D, Norris K, Martins D** 2005 The prevalence of hypovitaminosis D among US adults: data from the NHANES III. Ethn Dis 15:S5-97-101

4. **Holick MF** 1985 The photobiology of vitamin D and its consequences for humans. Ann N Y Acad Sci 453:1-13

5. **DeLuca HF** 1988 The vitamin D story: a collaborative effort of basic science and clinical medicine. FASEB J 2:224-236

6. **Holick MF** 2004 Vitamin D: importance in the prevention of cancers, type 1 diabetes, heart disease, and osteoporosis. Am J Clin Nutr 79:362-371

7. **Merke J, Milde P, Lewicka S, Hugel U, Klaus G, Mangelsdorf DJ, Haussler MR, Rauterberg EW, Ritz E** 1989 Identification and regulation of 1,25-dihydroxyvitamin D3 receptor activity and biosynthesis of 1,25-dihydroxyvitamin D3. Studies in cultured bovine aortic endothelial cells and human dermal capillaries. J Clin Invest 83:1903-1915

8. **Somjen D, Weisman Y, Kohen F, Gayer B, Limor R, Sharon O, Jaccard N, Knoll E, Stern N** 2005 25-hydroxyvitamin D3-1alpha-hydroxylase is expressed in human vascular smooth muscle cells and is upregulated by parathyroid hormone and estrogenic compounds. Circulation 111:1666-1671

9. **Merke J, Hofmann W, Goldschmidt D, Ritz E** 1987 Demonstration of 1,25(OH)2 vitamin D3 receptors and actions in vascular smooth muscle cells in vitro. Calcif Tissue Int 41:112-114

10. **Holick MF** 2006 High prevalence of vitamin D inadequacy and implications for health. Mayo Clin Proc 81:353-373

11. **Boonstra A, Barrat FJ, Crain C, Heath VL, Savelkoul HF, O'Garra A** 2001 1alpha,25-Dihydroxyvitamin d3 has a direct effect on naive CD4(+) T cells to enhance the development of Th2 cells. J Immunol 167:4974-4980

12. **Willheim M, Thien R, Schrattbauer K, Bajna E, Holub M, Gruber R, Baier K, Pietschmann P, Reinisch W, Scheiner O, Peterlik M** 1999 Regulatory effects of 1alpha,25-dihydroxyvitamin D3 on the cytokine production of human peripheral blood lymphocytes. J Clin Endocrinol Metab 84:3739-3744

13. **Rigby WF, Denome S, Fanger MW** 1987 Regulation of lymphokine production and human T lymphocyte activation by 1,25-dihydroxyvitamin D3. Specific inhibition at the level of messenger RNA. J Clin Invest 79:1659-1664

14. **O'Connell TD, Berry JE, Jarvis AK, Somerman MJ, Simpson RU** 1997 1,25-Dihydroxyvitamin D3 regulation of cardiac myocyte proliferation and hypertrophy. Am J Physiol 272:H1751-1758

15. **Inoue M, Wakasugi M, Wakao R, Gan N, Tawata M, Nishii Y, Onaya T** 1992 A synthetic analogue of vitamin D3, 22-oxa-1,25-dihydroxy-vitamin D3, stimulates the production of prostacyclin by vascular tissues. Life Sci 51:1105-1112

16. **Sigmund CD, Okuyama K, Ingelfinger J, Jones CA, Mullins JJ, Kane C, Kim U, Wu CZ, Kenny L, Rustum Y, et al.** 1990 Isolation and characterization of renin-expressing cell lines from transgenic mice containing a renin-promoter viral oncogene fusion construct. J Biol Chem 265:19916-19922

17. **Kimura Y, Kawamura M, Owada M, Oshima T, Murooka M, Fujiwara T, Hiramori K** 1999 Effectiveness of 1,25-dihydroxyvitamin D supplementation on blood pressure reduction in a pseudohypoparathyroidism patient with high renin activity. Intern Med 38:31-35

18. **Li YC, Kong J, Wei M, Chen ZF, Liu SQ, Cao LP** 2002 1,25-Dihydroxyvitamin D(3) is a negative endocrine regulator of the renin-angiotensin system. J Clin Invest 110:229-238

19. **Voors AW, Johnson WD** 1979 Altitude and arteriosclerotic heart disease mortality in white residents of 99 of the 100 largest cities in the United States. J Chronic Dis 32:157-162

20. **Fleck A** 1989 Latitude and ischaemic heart disease. Lancet 1:613

21. **Grimes DS, Hindle E, Dyer T** 1996 Sunlight, cholesterol and coronary heart disease. QJM 89:579-589

22. **Rostand SG** 1997 Ultraviolet light may contribute to geographic and racial blood pressure differences. Hypertension 30:150-156

23. **Resnick LM, Muller FB, Laragh JH** 1986 Calcium-regulating hormones in essential hypertension. Relation to plasma renin activity and sodium metabolism. Ann Intern Med 105:649-654

24. **Lind L, Hanni A, Lithell H, Hvarfner A, Sorensen OH, Ljunghall S** 1995 Vitamin D is related to blood pressure and other cardiovascular risk factors in middle-aged men. Am J Hypertens 8:894-901

25. **Kristal-Boneh E, Froom P, Harari G, Ribak J** 1997 Association of calcitriol and blood pressure in normotensive men. Hypertension 30:1289-1294

26. **Watson KE, Abrolat ML, Malone LL, Hoeg JM, Doherty T, Detrano R, Demer LL** 1997 Active serum vitamin D levels are inversely correlated with coronary calcification. Circulation 96:1755-1760

27. **Doherty TM, Tang W, Dascalos S, Watson KE, Demer LL, Shavelle RM, Detrano RC** 1997 Ethnic origin and serum levels of 1alpha,25-dihydroxyvitamin D3 are independent predictors of coronary calcium mass measured by electron-beam computed tomography. Circulation 96:1477-1481

28. **Scragg R, Jackson R, Holdaway IM, Lim T, Beaglehole R** 1990 Myocardial infarction is inversely associated with plasma 25-hydroxyvitamin D3 levels: a community-based study. Int J Epidemiol 19:559-563

29. **Poole KE, Loveridge N, Barker PJ, Halsall DJ, Rose C, Reeve J, Warburton EA** 2006 Reduced vitamin D in acute stroke. Stroke 37:243-245

30. **Wang TJ, Pencina MJ, Booth SL, Jacques PF, Ingelsson E, Lanier K, Benjamin EJ, D'Agostino RB, Wolf M, Vasan RS** 2008 Vitamin D deficiency and risk of cardiovascular disease. Circulation 117:503-511

31. **Vita JA, Keaney JF, Jr.** 2002 Endothelial function: a barometer for cardiovascular risk? Circulation 106:640-642

32. **Neunteufl T, Heher S, Katzenschlager R, Wolfl G, Kostner K, Maurer G, Weidinger F** 2000 Late prognostic value of flow-mediated dilation in the brachial artery of patients with chest pain. Am J Cardiol 86:207-210

33. **Gokce N, Keaney JF, Jr., Hunter LM, Watkins MT, Menzoian JO, Vita JA** 2002 Risk stratification for postoperative cardiovascular events via noninvasive assessment of endothelial function: a prospective study. Circulation 105:1567-1572

34. **Gokce N, Keaney JF, Jr., Hunter LM, Watkins MT, Nedeljkovic ZS, Menzoian JO, Vita JA** 2003 Predictive value of noninvasively determined endothelial dysfunction for long-term cardiovascular events in patients with peripheral vascular disease. J Am Coll Cardiol 41:1769-1775

35. **Suwaidi JA, Hamasaki S, Higano ST, Nishimura RA, Holmes DR, Jr., Lerman A** 2000 Long-term follow-up of patients with mild coronary artery disease and endothelial dysfunction. Circulation 101:948-954

36. **Higashi Y, Sasaki S, Sasaki N, Nakagawa K, Ueda T, Yoshimizu A, Kurisu S, Matsuura H, Kajiyama G, Oshima T** 1999 Daily aerobic exercise improves reactive hyperemia in patients with essential hypertension. Hypertension 33:591-597

37. **Ziccardi P, Nappo F, Giugliano G, Esposito K, Marfella R, Cioffi M, D'Andrea F, Molinari AM, Giugliano D** 2002 Reduction of inflammatory cytokine concentrations and improvement of endothelial functions in obese women after weight loss over one year. Circulation 105:804-809

38. **Celermajer DS, Sorensen KE, Georgakopoulos D, Bull C, Thomas O, Robinson J, Deanfield JE** 1993 Cigarette smoking is associated with dose-related and potentially reversible impairment of endothelium-dependent dilation in healthy young adults. Circulation 88:2149-2155

39. **Mather KJ, Verma S, Anderson TJ** 2001 Improved endothelial function with metformin in type 2 diabetes mellitus. J Am Coll Cardiol 37:1344-1350

40. **Anderson TJ, Meredith IT, Yeung AC, Frei B, Selwyn AP, Ganz P** 1995 The effect of cholesterol-lowering and antioxidant therapy on endothelium-dependent coronary vasomotion. N Engl J Med 332:488-493

41. **Mancini GB, Henry GC, Macaya C, O'Neill BJ, Pucillo AL, Carere RG, Wargovich TJ, Mudra H, Luscher TF, Klibaner MI, Haber HE, Uprichard AC, Pepine CJ, Pitt B** 1996 Angiotensin-converting enzyme inhibition with quinapril improves endothelial vasomotor dysfunction in patients with coronary artery disease. The TREND (Trial on Reversing ENdothelial Dysfunction) Study. Circulation 94:258-265

42. **Gocke N** 2002 Clinical manifestations of endothelial dysfunction. Philadelphia, PA: Lippincott Williams and Wilkins.

43. **Libby P, Ridker PM, Maseri A** 2002 Inflammation and atherosclerosis. Circulation 105:1135-1143

44. **Ludmer PL, Selwyn AP, Shook TL, Wayne RR, Mudge GH, Alexander RW, Ganz P** 1986 Paradoxical vasoconstriction induced by acetylcholine in atherosclerotic coronary arteries. N Engl J Med 315:1046-1051

45. **Laurent S, Boutouyrie P, Asmar R, Gautier I, Laloux B, Guize L, Ducimetiere P, Benetos A** 2001 Aortic stiffness is an independent predictor of all-cause and cardiovascular mortality in hypertensive patients. Hypertension 37:1236-1241

46. **Oliver JJ, Webb DJ** 2003 Noninvasive assessment of arterial stiffness and risk of atherosclerotic events. Arterioscler Thromb Vasc Biol 23:554-566

47. **Mathieu C, Adorini L** 2002 The coming of age of 1,25-dihydroxyvitamin D(3) analogs as immunomodulatory agents. Trends Mol Med 8:174-179

48. **Lemire JM** 1992 Immunomodulatory role of 1,25-dihydroxyvitamin D3. J Cell Biochem 49:26-31

49. **Hayes CE** 2000 Vitamin D: a natural inhibitor of multiple sclerosis. Proc Nutr Soc 59:531-535

50. **Mahon BD, Gordon SA, Cruz J, Cosman F, Cantorna MT** 2003 Cytokine profile in patients with multiple sclerosis following vitamin D supplementation. J Neuroimmunol 134:128-132

51. **Lange U, Jung O, Teichmann J, Neeck G** 2001 Relationship between disease activity and serum levels of vitamin D metabolites and parathyroid hormone in ankylosing spondylitis. Osteoporos Int 12:1031-1035

52. **Briffa NK, Keogh AM, Sambrook PN, Eisman JA** 2003 Reduction of immunosuppressant therapy requirement in heart transplantation by calcitriol. Transplantation 75:2133-2134

53. **Hullett DA, Cantorna MT, Redaelli C, Humpal-Winter J, Hayes CE, Sollinger HW, Deluca HF** 1998 Prolongation of allograft survival by 1,25-dihydroxyvitamin D3. Transplantation 66:824-828

54. **Timms PM, Mannan N, Hitman GA, Noonan K, Mills PG, Syndercombe-Court D, Aganna E, Price CP, Boucher BJ** 2002 Circulating MMP9, vitamin D and variation in the TIMP-1 response with VDR genotype: mechanisms for inflammatory damage in chronic disorders? QJM 95:787-796

55. **Van den Berghe G, Van Roosbroeck D, Vanhove P, Wouters PJ, De Pourcq L, Bouillon R** 2003 Bone turnover in prolonged critical illness: effect of vitamin D. J Clin Endocrinol Metab 88:4623-4632

56. **Schleithoff SS, Zittermann A, Tenderich G, Berthold HK, Stehle P, Koerfer R** 2006 Vitamin D supplementation improves cytokine profiles in patients with congestive heart failure: a double-blind, randomized, placebo-controlled trial. Am J Clin Nutr 83:754-759

57. **Zittermann A, Schleithoff SS, Koerfer R** 2006 Vitamin D insufficiency in congestive heart failure: why and what to do about it? Heart Fail Rev 11:25-33

58. **Jialal I, Devaraj S, Venugopal SK** 2004 C-reactive protein: risk marker or mediator in atherothrombosis? Hypertension 44:6-11

59. **Verma S** 2004 C-reactive protein incites atherosclerosis. Can J Cardiol 20 Suppl B:29B-31B

60. **Perez-Castrillon JL, Vega G, Abad L, Sanz A, Chaves J, Hernandez G, Duenas A** 2007 Effects of Atorvastatin on vitamin D levels in patients with acute ischemic heart disease. Am J Cardiol 99:903-905

61. **Grimes DS** 2006 Are statins analogues of vitamin D? Lancet 368:83-86

62. **London GM, Guerin AP, Verbeke FH, Pannier B, Boutouyrie P, Marchais SJ, Metivier F** 2007 Mineral metabolism and arterial functions in end-stage renal disease: potential role of 25-hydroxyvitamin D deficiency. J Am Soc Nephrol 18:613-620

63. **Bhalla AK, Amento EP, Clemens TL, Holick MF, Krane SM** 1983 Specific high-affinity receptors for 1,25-dihydroxyvitamin D3 in human peripheral blood mononuclear cells: presence in monocytes and induction in T lymphocytes following activation. J Clin Endocrinol Metab 57:1308-1310

64. **Provvedini DM, Tsoukas CD, Deftos LJ, Manolagas SC** 1983 1,25-dihydroxyvitamin D3 receptors in human leukocytes. Science 221:1181-1183

65. **Veldman CM, Cantorna MT, DeLuca HF** 2000 Expression of 1,25-dihydroxyvitamin D(3) receptor in the immune system. Arch Biochem Biophys 374:334-338

66. **van Etten E, Mathieu C** 2005 Immunoregulation by 1,25-dihydroxyvitamin D3: basic concepts. J Steroid Biochem Mol Biol 97:93-101

67. **Mahon BD, Wittke A, Weaver V, Cantorna MT** 2003 The targets of vitamin D depend on the differentiation and activation status of CD4 positive T cells. J Cell Biochem 89:922-932

68. **Bemiss CJ, Mahon BD, Henry A, Weaver V, Cantorna MT** 2002 Interleukin-2 is one of the targets of 1,25-dihydroxyvitamin D3 in the immune system. Arch Biochem Biophys 402:249-254

69. **Cantorna MT, Humpal-Winter J, DeLuca HF** 2000 In vivo upregulation of interleukin-4 is one mechanism underlying the immunoregulatory effects of 1,25-dihydroxyvitamin D(3). Arch Biochem Biophys 377:135-138

70. **Lefebvre d'Hellencourt C, Montero-Menei CN, Bernard R, Couez D** 2003 Vitamin D3 inhibits proinflammatory cytokines and nitric oxide production by the EOC13 microglial cell line. J Neurosci Res 71:575-582

71. **Peterlik M, Cross HS** 2005 Vitamin D and calcium deficits predispose for multiple chronic diseases. Eur J Clin Invest 35:290-304

72. **Froicu M, Weaver V, Wynn TA, McDowell MA, Welsh JE, Cantorna MT** 2003 A crucial role for the vitamin D receptor in experimental inflammatory bowel diseases. Mol Endocrinol 17:2386-2392

73. **Cantorna MT, Hayes CE, DeLuca HF** 1996 1,25-Dihydroxyvitamin D3 reversibly blocks the progression of relapsing encephalomyelitis, a model of multiple sclerosis. Proc Natl Acad Sci U S A 93:7861-7864

74. **Ross R** 1999 Atherosclerosis--an inflammatory disease. N Engl J Med 340:115-126

75. **Ikonomidis I, Andreotti F, Economou E, Stefanadis C, Toutouzas P, Nihoyannopoulos P** 1999 Increased proinflammatory cytokines in patients with chronic stable angina and their reduction by aspirin. Circulation 100:793-798

76. **Roth P, Stanley ER** 1992 The biology of CSF-1 and its receptor. Curr Top Microbiol Immunol 181:141-167

77. **Amento EP, Ehsani N, Palmer H, Libby P** 1991 Cytokines and growth factors positively and negatively regulate interstitial collagen gene expression in human vascular smooth muscle cells. Arterioscler Thromb 11:1223-1230

78. **Zwaka TP, Hombach V, Torzewski J** 2001 C-reactive protein-mediated low density lipoprotein uptake by macrophages: implications for atherosclerosis. Circulation 103:1194-1197

79. **Blaschke F, Bruemmer D, Yin F, Takata Y, Wang W, Fishbein MC, Okura T, Higaki J, Graf K, Fleck E, Hsueh WA, Law RE** 2004 C-reactive protein induces apoptosis in human coronary vascular smooth muscle cells. Circulation 110:579-587

80. **Li SH, Szmitko PE, Weisel RD, Wang CH, Fedak PW, Li RK, Mickle DA, Verma S** 2004 C-reactive protein upregulates complement-inhibitory factors in endothelial cells. Circulation 109:833-836

81. **van der Wal AC, Das PK, Tigges AJ, Becker AE** 1992 Adhesion molecules on the endothelium and mononuclear cells in human atherosclerotic lesions. Am J Pathol 141:1427-1433

82. **O'Brien KD, Allen MD, McDonald TO, Chait A, Harlan JM, Fishbein D, McCarty J, Ferguson M, Hudkins K, Benjamin CD, et al.** 1993 Vascular cell adhesion molecule-1 is expressed in human coronary atherosclerotic plaques. Implications for the mode of progression of advanced coronary atherosclerosis. J Clin Invest 92:945-951

83. **Johnson-Tidey RR, McGregor JL, Taylor PR, Poston RN** 1994 Increase in the adhesion molecule P-selectin in endothelium overlying atherosclerotic plaques. Coexpression with intercellular adhesion molecule-1. Am J Pathol 144:952-961

84. **Danesh J, Wheeler JG, Hirschfield GM, Eda S, Eiriksdottir G, Rumley A, Lowe GD, Pepys MB, Gudnason V** 2004 C-reactive protein and other circulating markers of inflammation in the prediction of coronary heart disease. N Engl J Med 350:1387-1397

85. **Ridker PM, Buring JE, Cook NR, Rifai N** 2003 C-reactive protein, the metabolic syndrome, and risk of incident cardiovascular events: an 8-year follow-up of 14 719 initially healthy American women. Circulation 107:391-397

86. **Ridker PM, Cook N** 2004 Clinical usefulness of very high and very low levels of C-reactive protein across the full range of Framingham Risk Scores. Circulation 109:1955-1959

87. **Cesari M, Penninx BW, Newman AB, Kritchevsky SB, Nicklas BJ, Sutton-Tyrrell K, Rubin SM, Ding J, Simonsick EM, Harris TB, Pahor M** 2003 Inflammatory markers and onset of cardiovascular events: results from the Health ABC study. Circulation 108:2317-2322

88. **Ridker PM, Rifai N, Stampfer MJ, Hennekens CH** 2000 Plasma concentration of interleukin-6 and the risk of future myocardial infarction among apparently healthy men. Circulation 101:1767-1772

89. **Rallidis LS, Zolindaki MG, Manioudaki HS, Laoutaris NP, Velissaridou AH, Papasteriadis EG** 2002 Prognostic value of C-reactive protein, fibrinogen, interleukin-6, and macrophage colony stimulating factor in severe unstable angina. Clin Cardiol 25:505-510

90. **Ridker PM, Rifai N, Pfeffer M, Sacks F, Lepage S, Braunwald E** 2000 Elevation of tumor necrosis factor-alpha and increased risk of recurrent coronary events after myocardial infarction. Circulation 101:2149-2153

91. **Tuomisto K, Jousilahti P, Sundvall J, Pajunen P, Salomaa V** 2006 C-reactive protein, interleukin-6 and tumor necrosis factor alpha as predictors of incident coronary and cardiovascular events and total mortality. A population-based, prospective study. Thromb Haemost 95:511-518

92. **Martinesi M, Treves C, d'Albasio G, Bagnoli S, Bonanomi AG, Stio M** 2008 Vitamin D derivatives induce apoptosis and downregulate ICAM-1 levels in peripheral blood mononuclear cells of inflammatory bowel disease patients. Inflamm Bowel Dis 14:597-604

93. **Martinesi M, Bruni S, Stio M, Treves C** 2006 1,25-Dihydroxyvitamin D3 inhibits tumor necrosis factor-alpha-induced adhesion molecule expression in endothelial cells. Cell Biol Int 30:365-375

94. **Chen SF** 1995 1 alpha, 25-Dihydroxyvitamin D3 decreased ICAM-1 and ELAM-1 expressions on pulmonary microvascular endothelial cells and neutrophil motivation. J Steroid Biochem Mol Biol 52:67-70

95. **Kaneko A, Suzuki S, Hara M, Mori J, Kumagai M, Yajima H, Yamashita K, Kakizawa T, Yamazaki M, Takeda T, Miyamoto T, Ichikawa K, Hashizume K** 1999 1,25-Dihydroxyvitamin D3 suppresses the expression of the VCAM-1 receptor, VLA-4 in human leukemic HL-60 cells. Biochem Biophys Res Commun 255:371-376

96. **Giulietti A, van Etten E, Overbergh L, Stoffels K, Bouillon R, Mathieu C** 2007 Monocytes from type 2 diabetic patients have a pro-inflammatory profile. 1,25-Dihydroxyvitamin D(3) works as anti-inflammatory. Diabetes Res Clin Pract 77:47-57

97. **Corretti MC, Anderson TJ, Benjamin EJ, Celermajer D, Charbonneau F, Creager MA, Deanfield J, Drexler H, Gerhard-Herman M, Herrington D, Vallance P, Vita J, Vogel R** 2002 Guidelines for the ultrasound assessment of endothelial-dependent flow-mediated vasodilation of the brachial artery: a report of the International Brachial Artery Reactivity Task Force. J Am Coll Cardiol 39:257-265

98. **Sorensen KE, Celermajer DS, Spiegelhalter DJ, Georgakopoulos D, Robinson J, Thomas O, Deanfield JE** 1995 Non-invasive measurement of human endothelium dependent arterial responses: accuracy and reproducibility. Br Heart J 74:247-253

99. **Gottdiener JS, Bednarz J, Devereux R, Gardin J, Klein A, Manning WJ, Morehead A, Kitzman D, Oh J, Quinones M, Schiller NB, Stein JH, Weissman NJ** 2004 American Society of Echocardiography recommendations for use of echocardiography in clinical trials. J Am Soc Echocardiogr 17:1086-1119

100. **Cruickshank K, Riste L, Anderson SG, Wright JS, Dunn G, Gosling RG** 2002 Aortic pulse-wave velocity and its relationship to mortality in diabetes and glucose intolerance: an integrated index of vascular function? Circulation 106:2085-2090

101. **Sutton-Tyrrell K, Najjar SS, Boudreau RM, Venkitachalam L, Kupelian V, Simonsick EM, Havlik R, Lakatta EG, Spurgeon H, Kritchevsky S, Pahor M, Bauer D, Newman A** 2005 Elevated aortic pulse wave velocity, a marker of arterial stiffness, predicts cardiovascular events in well-functioning older adults. Circulation 111:3384-3390

102. **Marketou ME, Zacharis EA, Nikitovic D, Ganotakis ES, Parthenakis FI, Maliaraki N, Vardas PE** 2006 Early effects of simvastatin versus atorvastatin on oxidative stress and proinflammatory cytokines in hyperlipidemic subjects. Angiology 57:211-218
