# Supplementary material for: A Prospective Randomized Controlled Trial of the Effects of Vitamin D Supplementation on Cardiovascular Disease Risk
Source: PLoS One. 2012 May 7;7(5):e36617. doi: 10.1371/journal.pone.0036617 (PMC3346736; doi:10.1371/journal.pone.0036617)
Supplement: Table S1 — Unadjusted Changes from Baseline after 4 Months Stratified by Baseline Vitamin D Level. (DOCX) [file pone.0036617.s003.docx]

**Supplementary Table 3. Unadjusted Changes from Baseline after 4 Months Stratified by Baseline Vitamin D Level**.

| **Change** | **Baseline Vitamin D < 30 ng/dL**  **(N=53)** | **Baseline Vitamin D > 30 ng/dL**  **(N=57)** | **P-value between groups*** |
| --- | --- | --- | --- |
| **25(OH) vitamin D (ng/mL)** | 4.1 (9.8) | 11.37 (11.4) | **<0.001** |
| **Glucose (mg/dL)** | 3.7 (11.8) | 2.0 (8.6) | 0.397 |
| **Total/HDL cholesterol ratio** | 0.1 (0.4) | 0.1 (0.4) | 0.659 |
| **C-reactive protein (mg/L)** | 0.4 (4.3) | 0.2 (1.6) | 0.707 |
| **Brachial artery diameter (cm)** | 0.000 (0.011) | 0.005 (0.011) | **0.018** |
| **Absolute FMD (cm)** | 0.004 (0.008) | -0.002 (0.011) | **0.004** |
| **Maximum relative FMD (%)** | 1.1 (2.4) | -0.5 (3.4) | **0.002** |
| **Heart rate (bpm)** | 1.7 (5.0) | 2.1 (6.0) | 0.773 |
| **Brachial SBP (mmHg)** | -1.6 (8.4) | -1.3 (10.8) | 0.867 |
| **Brachial DBP (mmHg)** | -0.2 (4.9) | -0.8 (4.6) | 0.564 |
| **Central SBP (mmHg)** | -1.2 (7.5) | -1.3 (9.2) | 0.970 |
| **Central DBP (mmHg)** | -1.0 (4.6) | -0.2 (4.9) | 0.456 |
| **Central pulse pressure (mmHg)** | -0.5 (9.5) | -1.0 (7.9) | 0.801 |
| **Pulse Wave Velocity (m/s)** | 0.3 (0.9) | -0.2 (1.0) | **0.030** |
| **Augmentation index (%)** | 2.1 (4.7) | 1.5 (6.9) | 0.703 |

*p-values are not adjusted for multiple comparisons; apparent between-groups differences are independent of treatment group (see Figure 2)

*All values are means (standard deviations)*

Abbreviations as in Table 1
